# Perinatal mental health in Singapore—prevalence, knowledge, attitudes, and practices

**DOI:** 10.3389/fmed.2025.1623596

**Published:** 2025-09-11

**Authors:** Phaik Ling Quah, Zi Xi Poo, Helen Yu Chen, Tze-Ern Chua, Lay Kok Tan, Kok Hian Tan

**Affiliations:** ^1^Division of Obstetrics & Gynecology, KK Women's and Children's Hospital, Singapore, Singapore; ^2^Duke-National University of Singapore Graduate Medical School, Singapore, Singapore; ^3^Department of Maternal Fetal Medicine, KK Women's and Children's Hospital, Singapore, Singapore; ^4^Department of Psychological Medicine, KK Women's and Children's Hospital, Singapore, Singapore

**Keywords:** perinatal mental health, maternal mental health, perinatal depression, perinatal anxiety, Edinburgh Postnatal Depression Scale (EPDS), knowledge, attitudes, and practices (KAP), help-seeking behavior, prevalence

## Abstract

**Introduction:**

There is limited data on perinatal depression and anxiety rates, as well as on knowledge, attitudes, and practices related to perinatal mental health (PMH), particularly in an urban Southeast Asian population.

**Methods:**

From September to December 2022, 446 antenatal and 150 postnatal participants completed a 38-item survey assessing depressive and anxiety symptoms, along with knowledge, attitudes, and practices regarding PMH. Probable depression was defined as an Edinburgh Postnatal Depression Scale (EPDS) score of ≥15 during pregnancy or ≥13 postnatal. Probable anxiety was defined using the EPDS anxiety subscale (EPDS-3A), which comprises items 3, 4, and 5, with a score of ≥5 indicating probable anxiety.

**Results:**

The rates of probable depression were 11.9% antenatally and 23.7% postnatally, while anxiety rates were 48.4% and 56.7%, respectively. Only 63.8% of participants could identify symptoms of mental health disorders, and just 24.1% had received assessment or education from healthcare professionals. Although most women (57.0%) would seek support from their spouse or partner, only 15.5% indicated willingness to consult healthcare professionals. Most participants supported the need for mental health education (89.5%), screening (87.4%), and tailored guidelines (94.5%) for perinatal women.

**Conclusion:**

High rates of probable depression and anxiety, coupled with low mental health literacy, highlight the urgent need for comprehensive PMH education and guidelines in Singapore.

## Highlights

- Routine mental health screening: Integrate antenatal and postnatal mental health screening to detect and address the high rates of depression and anxiety early.
- Enhance mental health literacy: Implement targeted education programs to improve symptom recognition and encourage professional help-seeking.
- Engage partners in care: Involve spouses or partners in mental health support to strengthen family-centered care during the perinatal period.

## Introduction

Perinatal mental health (PMH) is increasingly recognized as a significant global public health concern. The perinatal period—defined by the World Health Organization (WHO) as encompassing pregnancy and the first year after childbirth (1)—is a particularly vulnerable time for women. A 2018 systematic review estimated that ~10%−20% of women experience mental health disorders during this period (2). These disorders are concerning, not only because of their potential to affect the mother's long-term physical and mental wellbeing, but also due to their broader implications for the child, family, and society (3, 4). The impact of maternal depression and anxiety on fetal neurodevelopment (5–7), and their adverse consequences in the postnatal period (8–10) have been well documented.

A meta-analysis from high-income countries reported prevalence rates of 11% for antenatal depression, and 13% for postnatal depression (11). Another meta-analysis showed that the prevalence of maternal anxiety during pregnancy varies depending on the specific type of anxiety disorder studied, with estimates reaching up to 13% (12). The COVID-19 pandemic further intensified stressors affecting perinatal mental health. A systematic review and meta-analysis found that lockdown measures significantly increased mental health risks for pregnant and postnatal women (13), while comparative studies reported higher rates of perinatal depression during the pandemic compared to pre-pandemic periods (14–16). In Singapore, local data from the Postnatal Depression Intervention Programme similarly showed a statistically significant rise in screen-positive postnatal depression cases in 2021 (17). However, much of the existing evidence remains rooted in Western populations, with limited representation of Asian cohorts (11–13), underscoring the need for more context-specific data from diverse cultural settings.

Singapore is a multi-ethnic island city-state in Southeast Asia. As a highly developed and urbanized nation, it differs from many other Asian settings in terms of cultural practices, healthcare access, and societal norms (18). Culture plays a central role in shaping women's experiences during pregnancy and the postnatal period, influencing how emotional distress is perceived, expressed, and managed. Cultural beliefs and norms, particularly in Asian societies, can significantly affect attitudes toward mental health and influence help-seeking behaviors during this critical time. Compared to Western contexts, Asian perspectives on PMH often reflect lower awareness of the potential adverse effects of PMH conditions on both mother and child, as well as greater reluctance to seek mental health support (19, 20). Limited mental health literacy further compounds this issue and remains a key barrier to early screening and intervention (21). Despite the importance of addressing these challenges, there is a notable lack of evidence on maternal knowledge, attitudes, and help-seeking practices during the perinatal period in Asian populations.

This study aims to describe the prevalence of maternal depression and anxiety during pregnancy and the postnatal period, and to explore knowledge, attitudes, and practices related to PMH among a multicultural population of women in Singapore.

## Methods

### Study sample

Caregivers participating in this study were enlisted as part of the IPRAMHO survey on Integrated Maternal Perinatal Mental Health Care (IMUM). Survey data were collected using a convenience sampling method from eligible participants attending the outpatient Obstetrics & Gynecology Clinic at KK Women's and Children's Hospital (KKH) in Singapore. Eligible participants were Singaporean or permanent residents who were pregnant, and women up to 12 weeks' postnatal and possessed the ability to comprehend English. Before taking part, participants needed to provide witnessed verbal consent to the research team. Subsequently, they were given an electronic version of the survey through FormSG (https://form.gov.sg/) for completion. The research procedures for this study received formal approval for an exempt review from the SingHealth Centralized Institutional Review Board (CIRB Ref No.: 2022/2454). Clinical trial number: not applicable. All the authors and participants surveyed have provided consent for the publication of this manuscript.

### Data collection

The survey of this study was administered between September 2022 and December 2022 when Singapore was still in the Disease Outbreak Response System Condition (DORSCON) framework level Yellow due to the COVID-19 outbreak (22), and a total of *n* = 602 survey responses were collected. The survey comprised 38 items that included data collected on participants' demographics, 10 items from the Edinburgh Postnatal Depression Scale (EPDS) (23), and nine items regarding the knowledge, attitudes, and practices of mental health during pregnancy and the postnatal period. The survey questionnaire was developed and reviewed by members of the Perinatal Mental Health Guidelines on Depression and Anxiety Committee, who were responsible for the development of the Singapore Perinatal Mental Health Guidelines on Depression and Anxiety (17). The development of the survey questionnaire went through an expert review and thorough literature review which establishes content validity (see Supplementary Data File 1). The survey took 10–15 min in total duration.

### Assessment of probable depressive and anxiety during pregnancy and postnatal

Self-reported depressive and anxiety symptoms were assessed during pregnancy and up to 12 weeks postnatal using the Edinburgh Postnatal Depression Scale (EPDS), a widely validated 10-item screening tool for perinatal populations. A local Singapore cohort study has demonstrated good reliability of the EPDS, with a Cronbach's alpha of 0.82 (5). Participants with EPDS scores ≥15 during pregnancy or ≥13 postnatal, based on established normative values (24), were considered at high risk for probable depression.

Anxiety symptoms were assessed using the EPDS-3A, which comprises items 3, 4, and 5 of the EPDS. While two local unpublished studies found limited screening accuracy for perinatal anxiety, we conducted a preliminary analysis using the cut-off established by Smith-Nielsen et al., where a score of ≥5 demonstrated optimal sensitivity (70.9%), specificity (92.2%), and an AUC of 0.926—suggesting potential utility as a time-efficient screening tool for probable anxiety in both pregnant and postnatal women (25).

Prevalence rates of probable depressive and anxiety symptoms were calculated separately for antenatal and postnatal participants. Additionally, prevalence was examined by trimester (first: 0–12 weeks, second: 13–27 weeks, third: 28–40 weeks) and presented as Supplementary Data File 1.

### Maternal knowledge, attitudes, and practices of PMH and guideline recommendations

To gauge participants' understanding of PMH, the survey included five key questions: (1) “Are you aware that mental health disorders, such as anxiety and depression, can occur during pregnancy or postnatal?” (2) “Do you know the symptoms or signs of mental health disorders (anxiety or depression) to watch for during pregnancy or postnatal?” (3) “Do you believe that mental health disorders (anxiety and depression) can have adverse consequences on your pregnancy outcomes and your child's health outcomes after birth?” (4) “Do you consider lifestyle habits (diet, exercise, and sleep) important for mental health before, during pregnancy, or postnatal?” (5) “During your pregnancy and postnatal, were you assessed or educated about mental health disorders (anxiety and depression) by your primary obstetrician or other healthcare professionals?” Additionally, one question focused on participants' attitudes toward seeking help for PMH symptoms: “If you were experiencing symptoms of anxiety or depression during your pregnancy or postnatal, who would you seek help from?” The final three questions aimed to understand participants' perceptions of PMH: (1) “Do you believe there are positive benefits to providing mental health education for pregnant mothers and mothers postnatal?” (2) “Do you think there are positive benefits to implementing mental health screening for pregnant mothers and mothers' postnatal?” (3) “Do you believe that mental health guidelines for pregnant mothers and mothers postnatal would be useful?”

### Statistical analyses

In total, 602 women were recruited, of whom those with missing values for the gestational age during pregnancy or number of postnatal weeks (*n* = 6) were excluded, resulting in an analytical antenatal sample of 446 participants and postnatal sample of 150 participants for the calculation of prevalence rates.

Characteristics of all the 602 participants were presented with continuous datasets that were normally distributed are presented as the mean and standard deviation (SD), while frequencies and percentages were used to describe categorical variables between the antenatal and postnatal participants. In a supplementary analysis examining the comparison of categorical variables between probable depression and anxiety across trimesters in pregnancy, a chi-squared test was utilized. Statistically significant results were determined at 2-sided *p* < 0.05. All analyses were performed using STATA software version 18.0 (StataCorp, College Station, US).

## Results

### Caregiver characteristics

A total of 602 women participated in the IMUM study, and their characteristics were presented in Table 1. In summary, a majority of the women were of Chinese ethnicity (71.3%), employed (83.6%), with university-level education (69.9%), and approximately half were nulliparous (53.5%). On average the women were 32.0 (SD: 4.3) years old, with a pre-pregnancy BMI of 22.8 (SD: 4.8) kg/m^2^ (Table 2). Only 8.5% of the women had newborns with medical issues in their latest pregnancy, with 22.4% of them having previous pregnancies that ended in miscarriages, stillbirths, or early infant deaths. Among the majority of these women (72.8%), the most recent pregnancy was planned (Table 1). The average depression screening scores were 9.0 (SD: 4.8) and 9.5 (SD: 4.7) for antenatal and postnatal women, respectively. The average anxiety screening scores were 4.2 (SD: 2.1) and 4.5 (SD: 2.0) for antenatal and postnatal women, respectively.

**Table 1:** ^*^Baseline characteristics of the *n* = 446 antenatal and *n* = 150 postnatal study participants.

**Maternal characteristics**	**All participants (*n* = 602)**	**Antenatal (*n* = 446)**	**Postnatal (*n* = 150)**
**Ethnicity**			
Chinese	429 (71.3)	319 (71.5)	108 (72.0)
Malay	107 (17.8)	79 (17.7)	27 (18.0)
Indian	46 (7.6)	33 (7.4)	10 (6.7)
Others	20 (3.3)	15 (3.4)	5 (3.3)
Employed	503 (83.6)	367 (82.3)	131 (87.3)
**Education**			
Primary/secondary	22 (3.7)	17 (3.8)	4 (2.7)
Post-secondary	159 (26.4)	123 (27.6)	33 (22.0)
University	421 (69.9)	306 (68.6)	113 (75.3)
Nulliparous	322 (53.5)	268 (60.1)	53 (35.)
Age (years)	32.0 (4.3)	31.7 (4.4)	32.7 (4.0)
Pre-pregnancy BMI (kg/m^2^)	22.8 (4.5)	22.7 (4.2)	23.2 (4.8)
Gestational age (weeks)	–	22.8 (0.4)	–
Postnatal (weeks)	–	–	8.0 (3.8)
^**^Newborn health issues in recent delivery	51 (8.5)	4 (5.4)	27 (18.0)
^***^Adverse outcomes from previous pregnancy	135 (22.4)	100 (22.4)	35 (23.3)
Planned pregnancy	438 (72.8)	329 (73.8)	105 (70.0)
^#^Depression screening scores	–	9.0 (4.8)	9.5 (4.7)
^∧^Anxiety screening scores	–	4.2 (2.1)	4.5 (2.0)

^*^Mean (SD) for continuous variables or *n* (%) for categorical variables calculated for n = 596; n = 6 missing data on gestational weeks or postnatal weeks.

^**^This includes low birth weight, jaundice, congenital heart defects.

^***^Previous miscarriages, stillbirth or early infant death.

EPDS, Edinburgh postnatal depression scale.

^#^Calculated using items 1–10 from the EPDS.

^∧^Calculated using items 3–5 from the EPDS.

**Table 2:** Prevalence of probable antenatal and postnatal depression as assessed by EPDS.

^*^Participants (*n*=602)	**^#^Prevalence for probable depression**	**% (95% CI)**
Antenatal (*n* = 446)	High risk for probable depression (EPDS scores ≥15)	11.9% (95% CI: 8.9%−15.1%)
Postnatal (*n* = 150)	High risk for probable depression (EPDS scores ≥13)	27.3% (95% CI: 20.4%– 35.2%)

EPDS, Edinburgh postnatal depression scale.

^*^n = 6 missing data on gestational weeks or postnatal weeks.

^#^Calculated using items 1–10 from the EPDS.

### Prevalence of probable depression and anxiety

The proportions of probable antenatal and postnatal depression were 11.9% (95% CI: 8.9%−15.1%) and 23.7% (95% CI: 20.4%−35.2%), respectively (Table 2). For anxiety, the reported proportions were 48.4% (95% CI: 43.7%−53.2%) during the antenatal period, and 56.7% (95% CI: 48.3%−64.7%) during the postnatal period (Table 3). During pregnancy, the proportion of depression cases was 6.1% (95% CI: 1.4%−12.3%) in the first trimester, 12.6% (95% CI: 8.2%−18.7%) in the second trimester, and 13.8% (95% CI: 9.0%−19.2%) in the third trimester. Anxiety proportions were 46.3% (95% CI: 35.0%−57.8%) in the first trimester, 44.3% (95% CI: 37.2%−52.5%) in the second trimester, and 53.6% (95% CI: 45.2%−59.8%) in the third trimester. Statistical analysis showed no significant differences between trimesters (*p* > 0.05) (Supplementary Table 1).

**Table 3:** Prevalence of probable antenatal and postnatal anxiety as assessed by EPDS.

^*^Participants	**^∧^Prevalence for probable anxiety**	**% (95% CI)**
Antenatal (*n* = 446)	High risk for probable anxiety (EPDS scores ≥5)	48.4% (95% CI: 43.7%−53.2%)
Postnatal (*n* = 150)	High risk for probable anxiety (EPDS scores ≥5)	56.7% (95% CI: 48.3%−64.7%)

EPDS, Edinburgh postnatal depression scale.

*n* = 6 missing data on gestational weeks or postnatal weeks.

^∧^Calculated using items 3–5 from the EPDS.

### Knowledge, attitudes, and practices of perinatal mental health

Table 4 presents data on women's knowledge, attitudes, and practices regarding perinatal mental health. A significant majority (96.8%) recognized that mental health disorders can occur during pregnancy or after childbirth, with 94.2% acknowledging the influence of lifestyle habits on mental health. While 77.4% were aware of potential adverse effects on pregnancy and child health, only 63.8% identified the signs and symptoms to watch for. Notably, 24.1% reported having received assessment or education about mental health disorders from their primary obstetrician or other healthcare professionals. Regarding help-seeking behavior for anxiety or depression symptoms during or after pregnancy, ~57.0% indicated they would turn to their spouse or partner, whereas only 15.5% were willing to seek help from healthcare professionals. Participants overwhelmingly recognized the value of mental health education (89.5%) and screening (87.4%), with 94.5% emphasizing the importance of mental health guidelines tailored for pregnant and postnatal women.

**Table 4:** Knowledge, attitudes, and practices of *n* = 602 study participants on perinatal mental health.

**KAP question**	***n* (%)**
Are you aware that mental health disorders (i.e., anxiety and depression) can occur during pregnancy or/and post-pregnancy?	
Aware	583 (96.8)
Not aware	19 (3.2)
Do you know what are the symptoms or signs of mental health disorders (i.e., anxiety or depression) to look out for during pregnancy or post-pregnancy?	
Yes	384 (63.8)
No	40 (7.0)
Not sure	176 (29.2)
Do you think mental health disorders (i.e., anxiety and depression) have adverse consequences on your pregnancy outcomes, and your child's health outcomes after birth?	
Yes	466 (77.4)
No	27 (4.5)
Not sure	109 (18.1)
Do you think lifestyle habits (i.e., diet, exercise and sleep) are important for mental health before, during pregnancy or post-pregnancy?	
Yes	567 (94.2)
No	13 (2.2)
Not sure	22 (3.7)
During your pregnancy and post-pregnancy, did your primary obstetrician or any other health care professionals assess you or educate you about mental health disorders (i.e., anxiety and depression)?	
Yes	145 (24.1)
No	372 (61.8)
Not sure	85 (14.1)
If you were experiencing symptoms of anxiety or depression during your pregnancy or post-pregnancy, who would you seek for help?	
Spouse/partner	343 (57.0)
Friends	54 (9.0)
Family	57 (9.5)
Healthcare professional	93 (15.5)
None/I will handle it on my own	55 (9.0)
Do you think that there are positive benefits to having mental health education for pregnant mothers and mother's post-pregnancy?	
Yes	539 (89.5)
No	14 (2.3)
Not sure	49 (8.1)
Do you think there are positive benefits to have mental health screening for pregnant mothers and mothers' post-pregnancy?	
Yes	526 (87.4)
No	9 (1.5)
Not sure	67 (11.1)
Do you think mental health guidelines for pregnant mothers and mothers' post-pregnancy will be useful?	
Yes	569 (94.5)
No	5 (0.8)
Not sure	28 (4.7)

## Discussion

Our study found that approximately one in 10 pregnant women and one in four women within 12 weeks postnatal reported symptoms indicative of probable depression. Notably, an even greater proportion of women were identified as experiencing probable anxiety, both during pregnancy (48.4%) and in the postnatal period (56.7%).

We observed a postnatal depression prevalence of 27.3%, as screened using the EPDS (cut-off ≥13), based on data collected in 2022. This rate is significantly higher than those reported in earlier years—4.9% in 2019 and 6.5% in 2021 (16)—based on data from outpatient clinic screening programs. As expected, cohort studies tend to report higher prevalence rates, likely due to self-selection bias and increased psychological awareness among participants. For example, postnatal depression rates in local cohort studies were 10.4% in 2014 (26), 17.0% in 2018 (27), and 9.8% (28), all using the same EPDS cut-off of ≥13.

Similarly, the prevalence of antenatal depression (EPDS >15) in our study (11.9%) exceeded earlier reports—7.3% in 2016 (29) and 9.0% in 2019 (30). While prevalence estimates for perinatal depression vary widely across studies due to methodological differences, our findings appear consistent with global trends showing an increase in postnatal depression. For instance, prevalence in the US rose from 16.9% in 2019 to 18.1% in 2020 (15), while in the UK it increased from 10.3% in 2014 to 23.9% in 2020 (31). Both studies attributed this rise, at least in part, to the impact of the COVID-19 pandemic on maternal mental health. Furthermore, the overall higher prevalence of probable perinatal depression observed in this cohort compared to previous local estimates may reflect both sample characteristics and methodological factors. A relatively high proportion of participants reported adverse pregnancy outcomes, which is associated with increased psychological vulnerability during the perinatal period. Methodological differences may also have contributed. While many previous local studies administered depression screening only once, often in mid-pregnancy and at month-3 postpartum (10, 26, 28, 29), our study assessed participants at multiple time points throughout pregnancy and up to 12 weeks postpartum. This extended follow-up period may have captured late-onset antenatal symptoms and postpartum onset, resulting in a higher overall prevalence.

We also found higher rates of probable perinatal anxiety (antenatal: 48.4%; postnatal: 56.7%), as assessed using the EPDS-3A (25), compared to a 2018 meta-analysis that reported pooled rates of 15.2% during pregnancy and 15.0% postnatal (32). In Singapore, earlier studies using the State-Trait Anxiety Inventory (STAI) reported lower antenatal anxiety rates of 17–19% in 2016 (8) and 29.5% in 2018 (33). A meta-analysis conducted during the COVID-19 pandemic found that anxiety symptoms in pregnant women exceeded rates of depression, with a pooled antenatal anxiety prevalence of 40% across 26 studies, primarily from Asia (34). Our findings of 48.4% are consistent with this upward trend and further supported by other studies linking heightened anxiety to the pandemic context (34–36).

An increase in antenatal depression from the first to the third trimester was also observed, with both depression and anxiety peaking in the third trimester. This aligns with prior systematic reviews and meta-analyses suggesting higher rates in the second and third trimesters (37, 38). However, not all studies observed similar trends. In Kenya (39), the highest depression prevalence occurred during the early second trimester, while in Bangladesh (40), depressive symptoms declined as pregnancy progressed. These differences may stem from variations in EPDS cut-offs > 12 in Kenya and > 10 in Bangladesh—vs. > 15 in our study. Unlike depression, trends in antenatal anxiety were less consistent across trimesters, possibly due to the lack of standardized diagnostic criteria. A review found pooled anxiety rates of 18.2% (first trimester), 19.1% (second), and 24.6% (third) (32), with other studies showing mixed trimester trends (39, 41).

Despite high overall awareness of perinatal mental health (PMH) disorders (96.8%), fewer women were aware of the adverse effects on pregnancy outcomes and child development (77.4%). Additionally, only 63.8% could recognize symptoms of PMH disorders—comparable to the 68.2% found in a Chinese study on postnatal literacy (42). A systematic review of studies in Western countries similarly highlighted poor symptom recognition among postnatal women (19). Since awareness and recognition are critical for timely help-seeking (43), the discrepancy is noteworthy.

Our findings show that women were more likely to seek support from their spouse (57%) than from healthcare professionals (15.5%). This pattern is consistent with both Western (21) and Chinese studies (42), which found a general preference for informal sources of help. For example, Gong et al. reported only 9.3% of women preferred seeking help from professionals (44), while Huang et al. noted that “knowledge and beliefs about professional help” scored lowest among literacy items (42). Cultural beliefs play a significant role in shaping attitudes toward mental health and help-seeking behaviors in Singapore (45). Among Chinese individuals, the concept of “face” and the perception of mental health struggles as a moral failing may contribute to stronger stigma and less social tolerance (46, 47). In contrast, while some Malay Muslim participants attributed mental health issues to spiritual possession—potentially delaying access to appropriate care—they also demonstrated greater acceptance of persons with mental health issues, possibly due to Islamic teachings that emphasize compassion, acceptance, and the divine purpose behind human suffering (48). Thus, our findings of low help-seeking from healthcare professionals align with existing evidence that stigma—rooted in conservative values, shame, loss of “face,” and cultural reluctance to discuss psychological distress—remains a major barrier to mental health care in Asian societies compared to Western populations (49).

Healthcare provider support for PMH also appears suboptimal. A recent local survey of 55 doctors found that 83.7% lacked confidence in managing PMH concerns, and 65.5% did not routinely screen for them (50). Similar patterns were reported in India, where 89% of providers did not screen for perinatal depression and less than half were familiar with the appropriate tools (51). A systematic review across 43 studies in Western countries also revealed only moderate PMH literacy among healthcare providers, with limited knowledge of symptoms and screening tools (52). This deficiency, observed among both women and providers (50, 52), poses a major barrier to delivering adequate PMH care. Encouragingly, 85% of women in our study endorsed the benefits of education and screening, and 94.5% agreed that PMH guidelines would be helpful.

### Strengths and limitations

The study's strengths include a robust sample size (*n* = 602), which is comparable to previous local studies (10, 26, 29, 30). We also captured self-reported symptoms of both depression and anxiety during pregnancy and postnatal. Notably, this is the first study in Singapore to assess PMH literacy—including knowledge, attitudes, and help-seeking behaviors—among perinatal women.

However, several limitations must be acknowledged. First, we used the EPDS to screen for both depression and anxiety, which may limit comparability with studies using alternative tools such as the STAI (10, 26, 29). Second, the psychometric properties of the EPDS-3A for anxiety screening have not yet been validated in our local population. Therefore, the high prevalence of anxiety reported here warrants further investigation. Furthermore, there is low internal consistency (Cronbach's α = 0.40) of the 9-item KAP section of the survey. This may be attributed to the multidimensional nature of the KAP framework, the small number of items, and the inclusion of knowledge-based questions that do not reflect a single latent construct. Despite this, the items were developed by expert consensus to ensure content relevance and remain useful for capturing key aspects of perinatal mental health literacy and behaviors. Future work may involve separating the KAP domains into subscales, conducting factor analysis to explore dimensionality, or refining and expanding the item pool to improve internal consistency. Third, convenience sampling from the outpatient obstetrics clinic at KK Women's and Children's Hospital may reduce the generalizability of findings to the broader Singapore population. Finally, it is worth noting that the majority of participants in our study were of Chinese ethnicity, employed, and had attained university-level education, which may limit the generalizability of our findings to more diverse or less-advantaged Asian populations.

## Conclusion

In conclusion, our study highlights high rates of perinatal depression and anxiety, alongside suboptimal mental health literacy among women. These findings, together with insights from a recent survey of healthcare professionals (50), informed the development of the Singapore Perinatal Mental Health Guidelines on Depression and Anxiety (17). In parallel, initiatives have been introduced to strengthen public education and outreach, as well as to enhance training within primary healthcare settings.

## Data Availability

The raw data supporting the conclusions of this article will be made available by the authors, without undue reservation.
