# Supplementary material for: Perinatal mental health in Singapore—prevalence, knowledge, attitudes, and practices
Source: Front Med (Lausanne). 2025 Sep 11;12:1623596. doi: 10.3389/fmed.2025.1623596 (PMC12460404; doi:10.3389/fmed.2025.1623596)
Supplement: Supplementary file 1 [file Table_1.pdf]

Supplementary Table 1: Prevalence of probable antenatal depression and anxiety as assessed by EPDS according to trimesters of pregnancy in n=446 participants

Probable depression and anxiety	First trimester (n=82)	Second trimester (n=183)	Third trimester (n=181)	p value
#High risk for probable depression (EPDS scores ≥15), % (95 CI%)	6.1% (95% CI: 1.4%-12.3%)	12.6 (95%CI: 8.2%-18.7%)	13.8% (95% CI: 9.0-19.2%)	0.19
^High risk for probable anxiety (EPDS-3A score of ≥5), % (95 CI%)	46.3% (95% CI: 35.0%-57.8%)	44.3% (95%CI: 37.2%-52.5%)	53.6% (95% CI: 45.2%-59.8%)	0.19

EPDS: Edinburgh postnatal depression scale
Calculated using items 1-10 from the EPDS
^ Calculated using items 3-5 from the EPDS
