# Supplementary material for: Perinatal mental health in Singapore—prevalence, knowledge, attitudes, and practices
Source: Front Med (Lausanne). 2025 Sep 11;12:1623596. doi: 10.3389/fmed.2025.1623596 (PMC12460404; doi:10.3389/fmed.2025.1623596)
Supplement: Supplementary file 2 [file Data_Sheet_1.pdf]

Date: ________________________

Integrated Maternal Perinatal Mental Health Care (I-MUM)

As you are pregnant or have recently had a baby (up to the past 12 weeks postnatal), you are eligible to participate in our mental health survey.

This survey will help the researchers of KK Women's and Children's Hospital understand better how we can better support the mental health of mothers.

Please answer all questions as honestly and accurately as you can – this is not a test, and there are no wrong or right answers. Please note that this survey is anonymous and confidential with no personal identifiers.

1) If you are currently pregnant, please state how far along is your pregnancy? (Please indicate "NA" if this does not apply to you)

   ____________________ gestational weeks

2) If you had recently given birth, how many weeks postpartum are you? (Please indicate "NA" if this does not apply to you.)

   _____________________weeks

3) What is your ethnicity?

   Chinese ☐
   Malay ☐
   Indian ☐
   Others (please state) ____________

4) What is your highest educational level?

   University ☐
   Post-secondary ☐
   Primary or secondary ☐
   Others (please state) ____________

5) Do you work:

Full-time ☐

Part-time ☐

Currently not working ☐

6)If you are working, what is your current occupation?

____

7) What is your age?

______________ years

8) What was your weight before your pregnancy?

___________ kilograms

9) What is your current weight?

____________kilograms

10) What is your current approximate height?

_________ centimeters

11) How many children do you have (excluding this current pregnancy) ?

None ☐

1 child ☐

2 children ☐

3 children and above ☐

12) In your most recent delivery (i.e. your older child if you are pregnant, or this current delivery if you are postnatal), did you baby (or babies) have any health or medical problems at birth? Please indicate "NA" is none.

____

13) How many times have you been pregnant before (total number of pregnancies including miscarriages, termination, or early infant death). Please indicate “NA” if this is your first pregnancy.

____

14) How many of your previous pregnancies were miscarriages, stillbirth or early infant death? (Please indicate “0” if none).

____

15) Was your/most current pregnancy planned?

Yes ☐

No ☐

16) When was your last pregnancy, before this most current pregnancy (please give date of your last delivery/pregnancy loss if you had a previous pregnancy before this, including miscarriages or terminations).

____

17) Did you have fertility treatment with your current/most recent pregnancy?

Yes ☐

No ☐

18) Do you have any health problems or medical conditions not related to pregnancy? (If yes, please indicate your condition). Examples- Hypertension, diabetes, treatment of mental health issues, etc.? (If no, please indicate “NA”).

____

19) Do you feel like you are receiving/you will receive sufficient support from your spouse/family members/social network during your pregnancy journey or with the care of your newborn baby?

Yes ☐

No ☐

Not sure ☐

For the next 10 questions, please check the answer that comes closest to how you have felt **IN THE PAST 7 DAYS**, not just how you feel today. All items must be completed.

20) I have been able to laugh and see the funny side of things

As much as I always could ☐
Not quite so much now ☐
Definitely not so much now ☐
Not at all ☐

21) I have looked forward with enjoyment to things

As much as I ever did ☐
Rather less than I used to ☐
Definitely less than I used to ☐
Hardly at all ☐

22) I have blamed myself unnecessarily when things went wrong

Yes, most of the time ☐
Yes, some of the time ☐
Not very often ☐
No, never ☐

23) I have been anxious or worried for no good reason

No, not at all ☐
Hardly ever ☐
Yes, sometimes ☐
Yes, very often ☐

24) I have felt scared or panicky for no very good reason

☐
☐
☐
☐

Yes, quite a lot

Yes, sometimes

No, not much

No, not at all

25) Things have been getting on top of me

Yes, most of the time I haven’t been able to cope at all ☐

Yes, sometimes I haven’t been coping as well as usual ☐

No, most of the time I have coped quite well ☐

No, I have been coping as well as ever ☐

26) I have been so unhappy that I have had difficulty sleeping

Yes, most of the time ☐

Yes, sometimes ☐

Not very often ☐

No, not at all ☐

27) I have felt sad or miserable

Yes, most of the time ☐

Yes, quite often ☐

Not very often ☐

No, not at all ☐

28) I have been so unhappy that I have been crying

Yes, most of the time ☐

Yes, quite often ☐

Only occasionally ☐

No, never ☐

29) The thought of harming myself has occurred to me

Yes, quite often ☐

Sometimes ☐

Hardly ever ☐

Never ☐

The next 9 questions are about your knowledge, attitudes and practices of mental health during your pregnancy and post-pregnancy

30) Are you aware that mental health disorders (i.e., anxiety and depression) can occur during pregnancy or/and post-pregnancy?

    Aware ☐

    Not aware ☐

31) Do you know what are the symptoms or signs of mental health disorders (i.e., anxiety or depression) to look out for during pregnancy or post-pregnancy?

    Yes ☐

    No ☐

    Not sure ☐

32) Do you think mental health disorders (i.e., anxiety and depression) have adverse consequences on your pregnancy outcomes, and your child's health outcomes after birth?

    Yes ☐

    No ☐

    Not sure ☐

33) Do you think lifestyle habits (i.e., diet, exercise and sleep) are important for mental health before, during pregnancy or post-pregnancy?

    Yes ☐

    No ☐

    ☐

Not sure

34) During your pregnancy and post-pregnancy, did your primary obstetrician or any other health care professionals assess you or educate you about mental health disorders (i.e., anxiety and depression)?

Yes ☐

No ☐

Not sure ☐

35) If you were experiencing symptoms of anxiety or depression during your pregnancy or post-pregnancy, who would you seek for help?

Spouse/partner ☐

Friends ☐

Family ☐

Healthcare professional ☐

None of the above/I would handle it on my own ☐

36) Do you think that there are positive benefits to having mental health education for pregnant mothers and mother's post-pregnancy?

Yes ☐

No ☐

Not sure ☐

37) Do you think there are positive benefits to have mental health screening for pregnant mothers and mothers' post-pregnancy?

Yes ☐

No ☐

Not sure ☐

38) Do you think mental health guidelines for pregnant mothers and mothers' post-pregnancy will be useful?

Yes ☐

No ☐

Not sure ☐
